# Multicenter cohort study demonstrates more consolidation in upper lungs on initial CT increases the risk of adverse clinical outcome in COVID-19 patients

**DOI:** 10.7150/thno.46465

**Published:** 2020-04-27

**Authors:** Qian Yu, Yuancheng Wang, Shan Huang, Songqiao Liu, Zhen Zhou, Shijun Zhang, Zhen Zhao, Yizhou Yu, Yi Yang, Shenghong Ju

**Affiliations:** 1 Department of Radiology, Zhongda Hospital, School of Medicine, Southeast University, Nanjing, China; 2 Department of Critical Care Medicine, Zhongda Hospital, School of Medicine, Southeast University, Nanjing, China; 3 School of Electronics Engineering and Computer Science, Peking University, China; 4 Department of Computer Science, The University of Hong Kong, Hong Kong, China

**Keywords:** COVID-19 pneumonia, CT scan, risk factor, outcome

## Abstract

**Rationale**: Chest computed tomography (CT) has been used for the coronavirus disease 2019 (COVID-19) monitoring. However, the imaging risk factors for poor clinical outcomes remain unclear. In this study, we aimed to assess the imaging characteristics and risk factors associated with adverse composite endpoints in patients with COVID-19 pneumonia.

**Methods**: This retrospective cohort study enrolled patients with laboratory-confirmed COVID-19 from 24 designated hospitals in Jiangsu province, China, between 10 January and 18 February 2020. Clinical and initial CT findings at admission were extracted from medical records. Patients aged < 18 years or without available clinical or CT records were excluded. The composite endpoints were admission to ICU, acute respiratory failure occurrence, or shock during hospitalization. The volume, density, and location of lesions, including ground-glass opacity (GGO) and consolidation, were quantitatively analyzed in each patient. Multivariable logistic regression models were used to identify the risk factors among age and CT parameters associated with the composite endpoints.

**Results**: In this study, 625 laboratory-confirmed COVID-19 patients were enrolled; among them, 179 patients without an initial CT at admission and 25 patients aged < 18 years old were excluded and 421 patients were included in analysis. The median age was 48.0 years and the male proportion was 53% (224/421). During the follow-up period, 64 (15%) patients had a composite endpoint. There was an association of older age (odds ratio [OR], 1.04; 95% confidence interval [CI]: 1.01-1.06; *P* = 0.003), larger consolidation lesions in the upper lung (Right: OR, 1.13; 95%CI: 1.03-1.25, *P* =0.01; Left: OR,1.15; 95%CI: 1.01-1.32; *P* = 0.04) with increased odds of adverse endpoints.

**Conclusion**: There was an association of older age and larger consolidation in upper lungs on admission with higher odds of poor outcomes in patients with COVID-19.

## Introduction

The coronavirus disease 2019 (COVID-19) was first described as atypical pneumonia with unknown etiology in Wuhan, China. It was subsequently shown to be caused by a novel coronavirus similar to severe acute respiratory syndrome coronavirus (SARS-CoV) and was designated as SARS-CoV-2 1,2. Coronavirus outbreak has caused thousands of deaths globally 3.

Since its outbreak, there have been several studies on COVID-19 1,4-7. Elderly patients with underlying diseases often experience adverse events, including intensive care unit (ICU) admission, acute respiratory failure, or death 1,8,9. However, there is a need for more detailed information regarding the occurrence and the progression of COVID-19 pneumonia to severe and critical conditions to allow improved individual monitoring and evaluation.

Chest computed tomography (CT) has been used to monitor the clinical course of COVID-19 10,11. Radiographic features, including ground-glass opacity (GGO) and consolidation, are crucial evaluation aspects 10-15. Previous studies have reported on the characteristics and evolution of COVID-19. However, they were limited by small sample sizes and lack of quantitative radiographic characteristics; therefore, the imaging risk factors for poor clinical outcomes remain unclear. Moreover, we used an artificial intelligence (AI) system in quantitative analysis which showed productivity in disease diagnosis and control since epidemic outbreaks 16.

In this study, we aimed to report the quantitative imaging characteristics and risk factors for adverse composite endpoints, including admission to ICU, acute respiratory failure occurrence, and shock during hospitalization in patients with COVID-19 pneumonia in Jiangsu province, China.

## Methods

The study design was approved by the appropriate ethics review board (The Ethics Committee of Zhongda Hospital Affiliated to Southeast University [2020ZDSYLL013-P01 and 2020ZDSYLL019-P01]). The requirement for informed consent was waived due to the emergent pandemic.

### Participants

**Figure 1:**
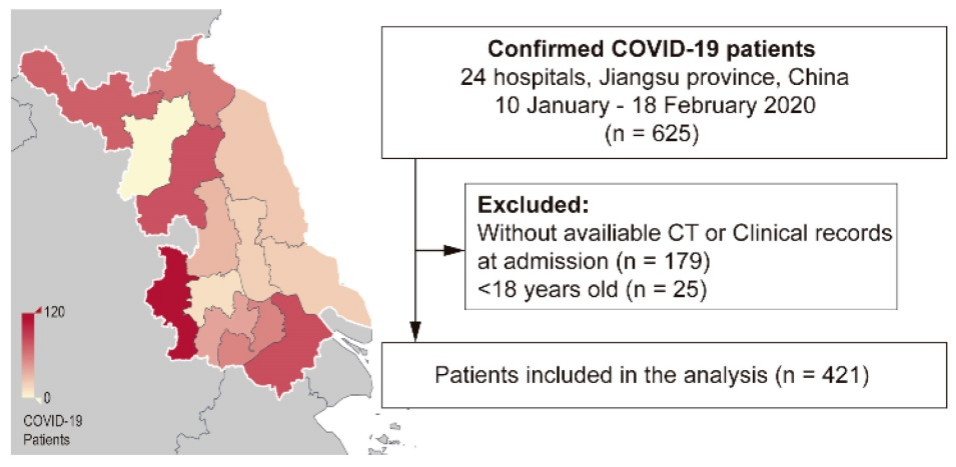
** Flowchart of the study.** Left: Heatmap of patients' distribution in Jiangsu province; Right: Study design. COVID-19: Coronavirus disease 2019

In this retrospective cohort study, we enrolled patients with laboratory-confirmed COVID-19 in Jiangsu province between 10 January and 18 February 2020. Laboratory confirmation was based on a positive result on high-throughput sequencing or real-time reverse-transcriptase-polymerase-chain-reaction (RT-PCR) assay of nasal and pharyngeal swab specimens 5. We extracted clinical and initial CT findings at admission from electronic medical records in the Department of Health, Jiangsu province. We excluded patients aged < 18 years old or with missing clinical or CT records on admission (Figure 1).

### Study outcomes

The primary composite endpoints were admission to ICU, acute respiratory failure occurrence, or shock during hospitalization, which have been used to assess the severity of COVID-19 or other infectious diseases 8,9,17. Acute respiratory failure was defined as oxygen saturation < 93%; partial pressure of oxygen in arterial blood < 60 mm Hg on room air; and/or requirement for high-flow nasal cannula therapy, non-invasive, or invasive mechanical ventilation. Patients meeting any of the following criteria: respiratory failure requiring mechanical ventilation; presence of shock; other organ failure that requires monitoring and treatment in the ICU were admitted to ICU treatment. However, patients presented respiratory failure or shock earlier than ICU admission or without ICU treatment were recorded as endpoint independently.

### CT imaging acquisition and AI evaluation

The CT examinations were performed according to standard non-contrast chest CT protocols in each institution. The CT scan details are shown in Supplementary Table S1. We quantitatively evaluated the volume, density, and location of lesions, including GGO and consolidation, in each patient. These characteristics were summarized as follows: the number of lung lobes involved; lesion volume percentage in each lobe and whole lung; Hounsfield units (HU) distribution within lesions; and distance from lesion to pulmonary pleurae (Table 2).

**Figure 2:**
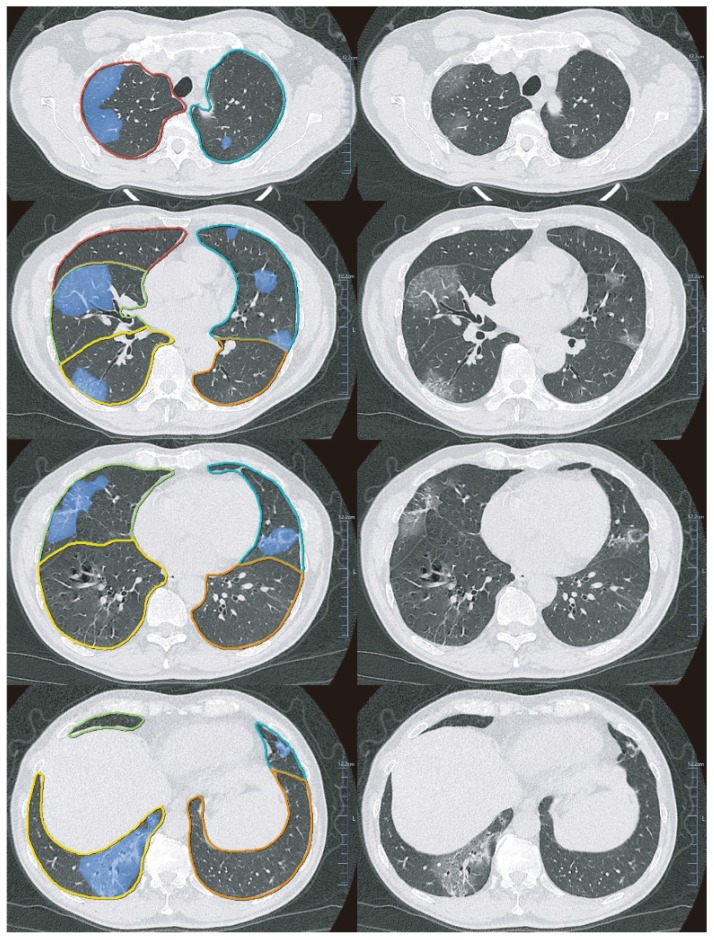
** Examples of Pulmonary lobe segmentation and opacity segmentation.** Left: Pulmonary lobes and opacities segmentation; Right: original images.

We performed lung segmentation, lesion extraction and labeling, and volume calculation using a dedicated multi-task deep learning algorithm developed for pulmonary pneumonia (Beijing Deepwise & League of PhD Technology Co.Ltd, China) 18. A 3D U-Net was introduced for pixel-level classification with a large-scale annotated data. The system's accuracy was guaranteed by engineering approach and manually checked by a radiologist (Y.C.W) with > 10 years of experience in chest imaging. Technical details and the accuracy of the AI method are showed in Supplementary Methods. Figure 2 shows an example of pulmonary lobe and opacities segmentation.

First, we segmented each lung lobe and the bilateral lungs and calculated the volume. Subsequently, we labeled the GGO and consolidation lesions in the corresponding lobes using the well-trained AI algorithm and computed the volume. Based on these parameters, we calculated the number of involved lung lobes and the lesion volume percentage in each lobe (right upper lobe [RUL], right middle lobe [RML], right lower lobe [RLL], left upper lobe [LUL], and left lower lobe [LLL]) and the whole lung. The RUL, RML, and LUL were defined as the upper lung while the remaining lobes were defined as the lower lung. Subsequently, to describe the HU distribution, we measured the volume of several opacities using the following cutoff HU values: -200, -400, and -600 HU, and calculated the percentage of each HU subsection volume in the whole lesions. We described the lesion location and distribution as the median distance from every lesion voxel to pulmonary pleurae in the upper and lower lung with a lower distance indicating subpleural area distribution.

### Statistical analysis

We reported continuous variables as the mean (standard deviation [SD]) or median (interquartile range [IQR]). We used Student's t-test or Mann-Whitney U test to compare between-group differences (presence and non-presence of primary composite endpoints) based on distributions. Categorical variables were presented as n (%) and compared using χ2 or Fisher's exact test.

We used multivariable logistic regression models to identify CT risk factors for the primary composite endpoints. Regarding clinical features, studies on COVID-19 have shown that age is a clear risk factor for death or adverse clinical outcomes 8. Therefore, we included age and the CT parameters into the multivariable logistic regression model. We performed a sensitivity analysis on the completed cases.

We set statistical significance at the 2-tailed *P* < 0.05. All analyses were performed using SPSS (version 22.0. IBM Crop. Armonk, NY, USA).

## Results

We enrolled 625 patients with laboratory-confirmed COVID-19 from 24 designated hospitals in Jiangsu province between 10^th^ January and 18^th^ February 2020 (Figure 1). We excluded 179 patients without initial CT scans or clinical records at admission and 25 patients aged < 18 years. Consequently, we included 421 patients in the final analysis. Among the included patients, 364/421 (86%) had a clear COVID-19 exposure history while 168/421 (46%) were imported from Wuhan. The median age was 48.0 years (IQR 34.0-58.0) and the male proportion was 53% (224/421). Fever was the most common symptom on admission (70% [294/421]), followed by coughing. Moreover, 89 (21%) patients had comorbidities on admission with hypertension being the most common (17% [70/421]).

Upon hospital admission, epidemiological, clinical, and laboratory data were collected with the median time from admission to the initial CT scan being 0 days (IQR: 2 days). The median time from illness onset to hospital admission was 5 days (IQR: 6 days) with no statistical difference between patients with or without clinical outcome. The median time from admission to a primary composite endpoint was 2 days (IQR: 0 days; range: 2-11 days). Until 6^th^ March 2020, there were no deaths and 350 (83%) patients were discharged.

During the 14-day follow-up period, 64/421 (15%) patients reached a primary composite endpoint. As shown in Table 1, compared with patients who did not reach an endpoint, those that did were older (57.2 [SD, 14.6] vs. 45.0 [SD, 14.9] years, *P* < 0.001) and had a higher fever prevalence (53/64 [83%] vs. 241/357 [68%], *P* = 0.02). Patients with an endpoint had a higher comorbidity proportion, including hypertension (20/64 [31%] vs. 50/357 [14%]), coronary heart disease (4/64 [6%] vs. 3/357 [1%]), and coronary heart disease (4/64 [6%] vs. 3/357 [1%]). Moreover, they had reduced white blood cell, lymphocyte, and platelet counts and increased C-reactive protein and D-dimer levels.

Initial CT findings revealed that 411 patients had pneumonia opacities; moreover, the 10 patients without opacities on initial CT findings did not present an endpoint during hospitalization. As shown in Table 2, CT quantitative evaluation of pulmonary lesions revealed that GGO and consolidation lesions in patients with endpoints occupied more lung lobes than those in patients without. Compared with patients without endpoints, those with endpoints had a higher percentage of lesions in the bilateral lungs, as well as larger GGO and consolidation lesions in each lung lobe. CT density of the lesion was largely distributed in the range of -200 to -600 HU and < -600 HU in patients with and without an endpoint, respectively. Peripheral distribution in all the patients was observed generally. Compared to patients without an endpoint, those with an endpoint showed lesions with a shorter distance to pulmonary pleurae.

**Figure 3:**
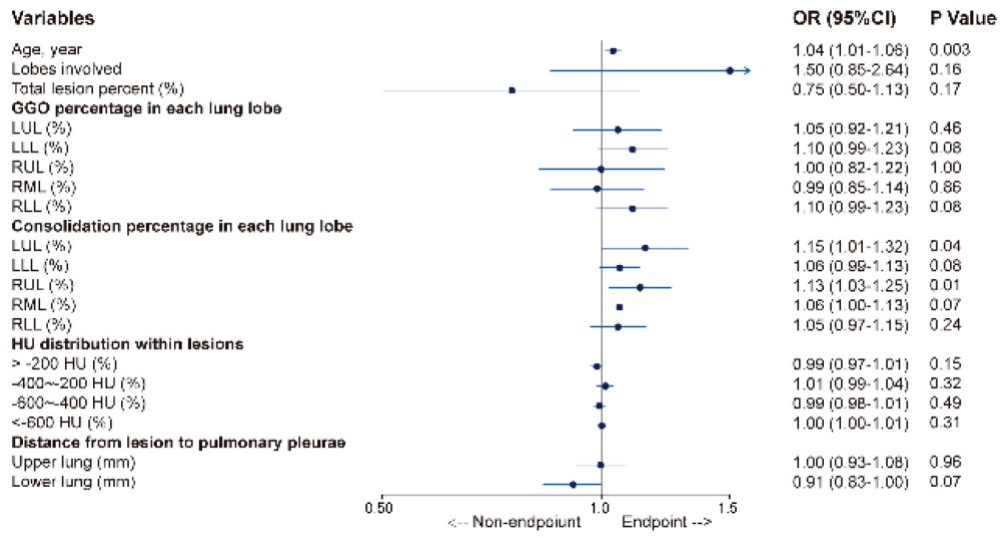
** Multivariable logistic regression to identify CT factors associated with composite endpoint in patients with COVID-19.** Abbreviations: RUL: right upper lobe; RML: right middle lobe; RLL: right lower lobe; LUL: left upper lobe; LLL: left lower lobe; HU: Hounsfield units.

Finally, 359 patients with complete data (on age and 18 CT parameters) were included in the multivariable logistic regression model. As shown in Figure 3, older age (odds ratio [OR], 1.04; 95% confidence interval [CI: 1.01-1.06; *P* =0.003), larger consolidation lesions in the RUL (OR, 1.13; 95% CI: 1.03-1.25; *P* = 0.01) and LUL (OR, 1.15; 95% CI: 1.01-1.32; *P* =0.04) were associated with increased odds of endpoints.

Sensitivity logistic regression analysis with only above 3 significant variables on the completed cases showed that the 3 variables remained significance related to the composite endpoint (*P*<0.05).

**Figure 4:**
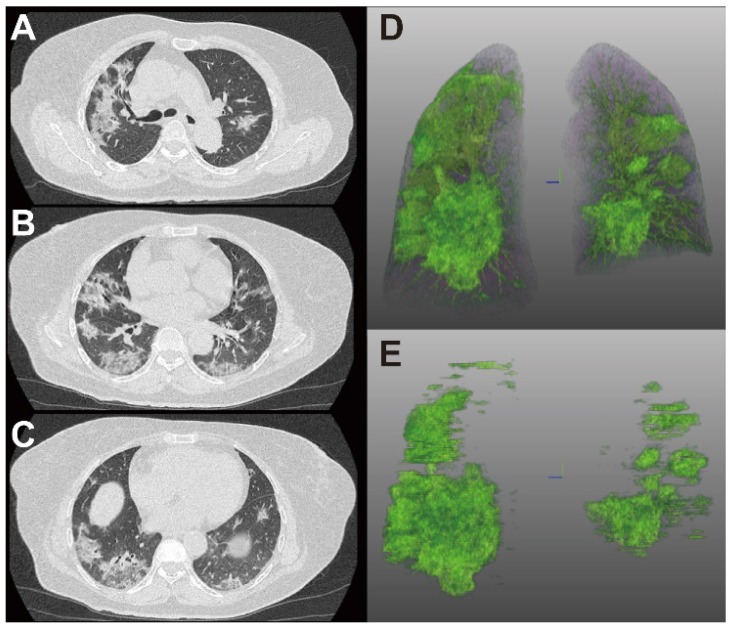
** 65-year-old woman with coronavirus disease 2019.** A-C. Non-contrast CT was performed on day of admission. D. Three-dimensional volume-rendered reconstruction shows the distribution of the opacities. E. Pulmonary opacities segmented by AI system. The patient had history of diabetes and hypertension and showed fever at admission. Patient developed acute respiratory failure at the third day of hospitalization. Lung CT images showed large areas of bilateral consolidation and ground-glass opacities, specifically in the upper lungs.

**Figure 5:**
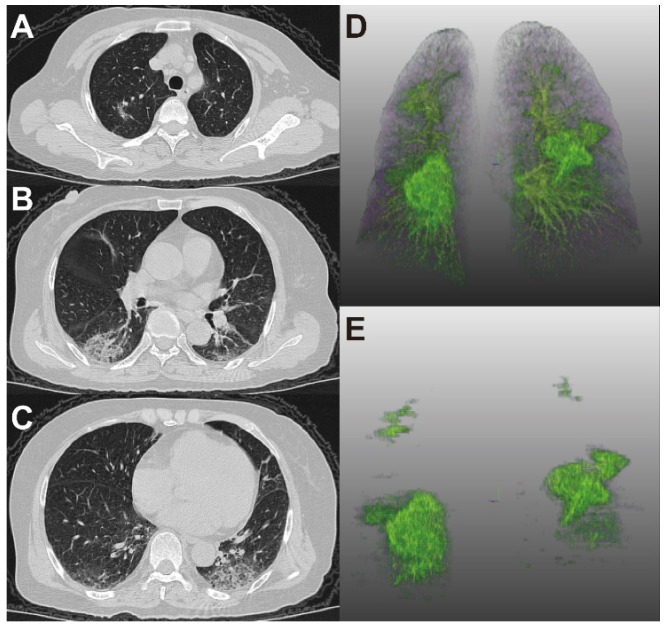
** 66-year-old woman with coronavirus disease 2019.** A-C. Non-contrast CT was performed on day of admission. D. Three-dimensional volume-rendered reconstruction shows the distribution of the opacities. E. Pulmonary opacities segmented by AI system. The patient showed fever and cough at admission. Patient did not reach clinical endpoint during hospitalization. Lung CT images showed that consolidation and ground-glass opacities mainly distributed in the lower lungs.

Figure 4 and 5 showed two typical cases with and without clinical endpoint during hospitalization.

## Discussion

In this retrospective, cohort, and AI-assisted study, we reported the imaging risk factors associated with adverse clinical composite endpoints, including admission to ICU, acute respiratory failure, and shock during hospitalization in patients with COVID-19 pneumonia in Jiangsu province, China. Generally, patients with adverse endpoints had worsened pulmonary conditions. Specifically, older age, larger consolidation lesions in upper lungs, and lesions closer to pulmonary pleurae in lower lungs on admission were associated with higher odds of adverse composite endpoints.

Our sample comprised of 421 patients with COVID-19 from whole Jiangsu province; among them, 364 (86%) had a clear COVID-19 exposure history while 46% (168/421) were from Wuhan. Given the combination of local and Wuhan cases, our findings could be generalizable to patients in other countries and regions where the epidemic has developed.

In our study, 64/421 (15%) patients had primary composite endpoints during hospitalization, which is less severe than that reported in patients in Wuhan 6. A previous study reported that 50/191 (26%) patients in Wuhan were admitted to ICU and 103/191 (54%) developed respiratory failure 8. We found an association of older age with the development of adverse composite endpoints, which is consistent with previous findings of increasing age, immunity defects 19, and more comorbidities 1 were associated with poor outcome during COVID-19 course.

In our study, we reported the pneumonia lesion component, amount and distribution associated with poor outcome quantitatively. And based on a large population, the results and conclusions may be considered specific and robust to patients with COVID-19 in clinical practice. Consolidation in upper lungs on initial CT at admission were risk factors associated with adverse endpoints after adjusting for age. Firstly, more consolidation has been reported in patients died from COVID-19 5. Histological examinations of lung biopsy samples from patients with COVID-19 have demonstrated bilateral diffuse alveolar damage with proteinaceous and fibrous exudates, which attributed to imaging appearance on chest CT 20,21. In patients with unfavorable outcome, consolidation development accelerated, which happened in all lobes, especially in the upper lungs.

**Figure 6:**
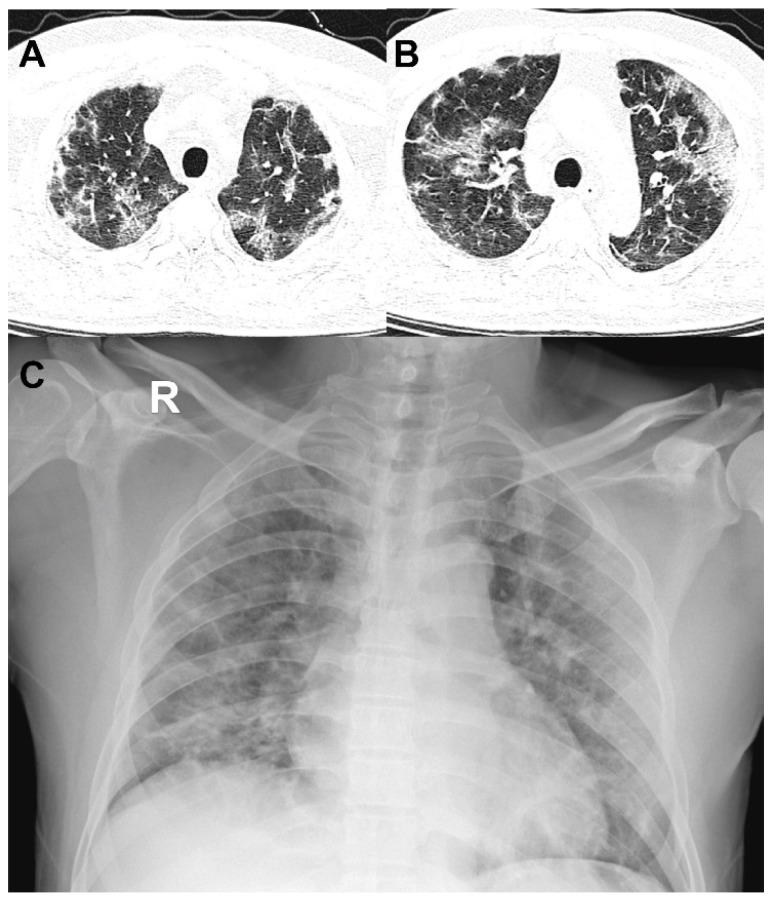
** 52-year-old man with coronavirus disease 2019.** A-B. Non-contrast CT was performed on day of admission. C. Chest X-ray was performed on day of admission. Patient developed acute respiratory failure at the second day of hospitalization. Chest CT images showed large areas of bilateral consolidation and ground-glass opacities in upper lungs and lesions showed peripheral distribution. Consist with CT, chest X-ray also showed patchy consolidation in bilateral lung periphery.

Secondly, in terms of distribution, the upper lung played an important role in risk evaluation for COVID-19 patients. Previous studies reported worse symptoms related to more pulmonary lesions 10,13,15,23. However, no detailed distribution information was described. Our study of 421 COVID-19 patients suggested an increased pulmonary lesion in bilateral upper lungs were independent risk factors for adverse clinical outcomes, which as far as we know, has not been reported. This finding is of significant clinical implications, not only in providing us a simpler way to identify high risk patients on CT, but also has potential to extend to chest radiographs, where upper lobe consolidations are easy to be perceived. (Figure 6).

Apart from consolidation, GGO is also the most common CT characteristics in COVID-19 pneumonia 14,15. Previous findings showed that there was GGO at the early disease stage, which showed an increasing followed by a decreasing growth trend, while consolidation development was delayed and after the GGO in general 10,22,23. We observed a positive correlation of the GGO in lower lungs and poor outcome. And GGO lesions on admission have been reported in severe cases of other respiratory viral diseases, including SARS and Middle East respiratory syndrome 24-26.

Additionally, our study revealed there was a negative correlation of the distance to pleurae with diffuse peripheral distribution in lower lungs (OR: 0.91, 95%CI 0.83-1.0; *P* = 0.07) showing deterioration signs related to adverse endpoints. Subpleural distribution is another common finding in COVID-19 pneumonia. Here, we measured the distance from every lesion voxel to pulmonary pleurae as a description of the lesion location and distribution. We observed generally peripheral distribution in all the patients, which is consistent with previous reports 14,15,23.

Finally, our study presented additional value of AI in COVID-19 researches apart from construction of diagnosis models 16, 27. AI played a significant role in this study and showed potential in COVID-19 diagnosis and patient monitoring. Based on high accuracy of lung and lesion segmentation from AI system, we measured the precise volume of opacities with different components, multiple HU blocks and location respectively. This method is superior to those studies based on visual evaluation 22-24 which may vary from person to person. The accuracy of lesion extraction is critical for risk factor identification in multivariate regression in our study. Moreover, the process of segmentation by AI only takes few minutes per patient making large scale quantification analysis possible.

This study has several limitations. First, given its retrospective observational design, there is a possibility of measurement and information bias. Second, although we analyzed imaging risk factors, we did not analyze the effects of other previously reported clinical characteristics associated with death or other fatal endpoints. Third, we assessed quantitative CT characteristics; however, we did not extract high-throughput imaging features, including histogram and texture. Moreover, the risk factors might have been affected by unadjusted confounders or unmeasured factors. Therefore, future studies should consider clinical factors, as well as CT features and deep radiological features.

## Conclusions

To our knowledge, this is the largest retrospective, cohort, and AI-assisted study on patients with COVID-19 to report on imaging risk factors associated with a clinical outcome. We found that older age and larger consolidation in upper lungs on admission were associated with higher odds of adverse composite endpoints, including admission to ICU, acute respiratory failure, or shock during hospitalization. The finding provided us a simpler way to identify high risk patients on CT that the elder with large consolidation in upper lungs should be paid more attention during the treatment of COVID-19. Initial CT evaluation and identification of these risk factors could be helpful toward the monitoring of COVID-19.

## Supplementary Material

Supplementary materials and methods, table.

## Figures and Tables

**Table 1:** Demographic, clinical, and laboratory characteristics of patients on admission

	Composite endpoint, total, mean (SD) or total, median (IQR), or n/total (%)			
Characteristics	All (N = 421)	Presence of endpoint (N = 64)	Non-presence of endpoint (N = 357)	*P*-value
Time from illness onset to hospital admission (day)	386,5(6)	60,6(5)	326,4(6)	0.10
Age (year)	421,46.9(15.4)	64,57.2(14.6)	357,45.0(14.9)	<0.001
Male	224/421(53%)	41/64(64%)	183/357(51%)	0.08
Current smoke	13/421(3%)	0/64(0%)	13/357(4%)	0.2
**Initial symptoms**				
Fever^a^	294/421(70%)	53/64(83%)	241/357(68%)	0.02
Cough	236/421(56%)	42/64(66%)	194/357(54%)	0.1
Sputum	107/421(25%)	21/64(33%)	86/357(24%)	0.2
Dyspnoea	2/421(0.5%)	1/64(2%)	1/357(0.3%)	0.3
Diarrheal	27/420(6%)	4/63(6%)	23/357(6%)	>0.99
**Comorbidity**				
Hypertension	70/421(17%)	20/64(31%)	50/357(14%)	0.002
Coronary heart disease	7/421(2%)	4/64(6%)	3/357(1%)	0.01
Cardiac dysfunction III-IV	2/421(0.5%)	1/64(2%)	1/357(0.3%)	0.3
Liver dysfunction^b^	2/421(0.5%)	1/64(2%)	1/357(0.3%)	0.3
Diabetes	26/421(6%)	9/64(14%)	17/357(5%)	0.01
Chronic kidney disease	4/421(1%)	1/64(2%)	3/357(2%)	0.5
Malignant tumor	5/421(1%)	1/64(2%)	4/357(1%)	0.6
Stoke	3/421(0.7%)	1/64(2%)	2/357(0.6%)	0.4
**Laboratory parameters**				
WBC Count (10^9^/L)	350,4.8(2.2)	58,4.2(2.2)	292,4.9(2.2)	0.03
Neutrophil (10^9^/L)	346,2.9(1.8)	58,2.8(2.1)	288,2.9(1.6)	0.9
Lymphocyte (10^9^/L)	344,1.3(0.8)	58,0.8(0.4)	286,1.4(0.8)	<0.001
Hemoglobin (g/L)	349,134.0(29.0)	58,138.0(25.2)	291,133.0(30.0)	0.6
Platelet (10^9^/L)	332,182.0(64.8)	53,155.0(64.5)	279,189.0(63.0)	<0.001
C-reactive protein (mg/L)	325,10.0(22.74)	51,31.0(67.1)	274,10.0(18.6)	<0.001
D-dimer (mg/L)	334,0.3(0.3)	57,0.4(0.8)	277,0.3(0.3)	0.006
**Outcome**				
Discharge from hospital	350/421(83%)	49/64(77%)	301/357(84%)	0.1
Death	0/421(0%)	0/64(0%)	0/357(0%)	-

a. Fever was defined as axillary temperature of at least 37.3°C. b. Liver dysfunction included cirrhosis, hepatic encephalopathy, and portal hypertension. Abbreviations: WBC: white blood cell; IQR: interquartile range; SD: standard deviation

**Table 2:** Radiological findings of patients on admission

	Composite endpoint, total, mean (SD) or total, median (IQR), or n/total (%)			
Characteristics	All(N=421)	Presence of endpoint (N=64)	Non-presence of endpoint (N=357)	*P*-value
Lobes involved	421,4(2)	64,5(0)	357,4(2)	< 0.001
Total lesion percent (%)	421,5.7(9.1)	64,16.1(5.4)	357,3.8(5.4)	< 0.001
**GGO percentage in each lung lobe**				
LUL (%)	421,0.8(3.7)	64,2.1(7.0)	357,0.6(2.7)	0.04
LLL (%)	421,1.1(5.2)	64,2.1(9.0)	357,0.9(4.2)	0.70
RUL (%)	421,0.5(1.8)	64,1.0(2.6)	357,0.4(1.5)	0.008
RML (%)	421,0.4(2.1)	64,0.8(3.6)	357,0.4(1.7)	0.25
RLL (%)	421,0.8(3.0)	64,1.2(5.6)	357,0.8(2.2)	0.16
**Consolidation percentage in each lung lobe**				
LUL (%)	421,2.9(7.0)	64,10.6(13.6)	357,1.5(3.4)	< 0.001
LLL (%)	421,7.5(14.0)	64,20.2(22.0)	357,5.2(10.6)	< 0.001
RUL (%)	421,3.9(9.5)	64,14.3(18.6)	357,2.0(4.8)	< 0.001
RML (%)	421,2.9(8.2)	64,10.0(15.6)	357,1.6(5.0)	< 0.001
RLL (%)	421,8.6(14.2)	64,21.9(23.3)	357,6.2(10.2)	< 0.001
**Density: HU distribution within lesions**				
> -200 HU (%)	421,12.1(9.8)	64,12.2(7.5)	347,11.7(10.2)	0.3
-400~-200 HU (%)	421,13.0(7.1)	64,15.1(6.3)	347,12.3(7.4)	0.004
-600~-400 HU (%)	421,20.4(7.0)	64,22.2(6.2)	347,20.0(7.1)	0.005
<-600 HU (%)	421,54.5(18.1)	64,50.5(15.0)	347,55.3(18.5)	0.036
**Location: Distance from lesion to pulmonary pleurae**				
Upper lung (mm)	369,14.1(6.7)	64,14.2(4.7)	305,14.1(7.1)	< 0.001
Lower lung (mm)	399,11.6(6.0)	63,10.4(3.8)	336,11.9(6.3)	< 0.001

Abbreviations: RUL: right upper lobe; RML: right middle lobe; RLL: right lower lobe; LUL: left upper lobe; LLL: left lower lobe; HU: Hounsfield units.

## Abbreviations

CT: Computed tomography
COVID-19: Coronavirus disease 2019
SARS-CoV: Severe acute respiratory syndrome coronavirus
ICU: Intensive care unit
GGO: Ground-glass opacity
RT-PCR: Reverse-transcriptase-polymerase-chain-reaction
RUL: Right upper lobe
RML: Right middle lobe
RLL: Right lower lobe
LUL: Left upper lobe
HU: Hounsfield units
SD: Standard deviation
