# Supplementary material for: Multicenter cohort study demonstrates more consolidation in upper lungs on initial CT increases the risk of adverse clinical outcome in COVID-19 patients
Source: Theranostics. 2020 Apr 27;10(12):5641–8. doi: 10.7150/thno.46465 (PMC7196305; doi:10.7150/thno.46465)
Supplement: Supplementary file 1 — Supplementary materials and methods, table. [file thnov10p5641s1.pdf]

Supplemental Materials

Materials and Methods

Artificial Intelligence (AI) method in imaging analysis: Pulmonary Lobe Segmentation

The proposed method could output pulmonary segment, lobe segment at the same time, by multi-task learning, which could lower the learning difficulties and enhance the performance of each task. 3D U-Net was used as the backbone network structure. Weak labels were annotated by drawing a box within each pulmonary segment to provide enough data for training, which reduced the need for precise annotations. Therefore, amount of weak labeled data was available for training within a short period. Besides, an active learning manner was adapted to choose the most informative samples for annotation and training, which highly improved the training efficiency.

Artificial Intelligence (AI) method in imaging analysis: Pulmonary Opacity Detection and Segmentation

The proposed method was designed to automatically detect and segment the pneumonia related symptom regions, like ground-glass opacity and consolidation. Feature pyramid network was used as the backbone network to achieve good performance when detecting different symptoms with various scales. Images with different window width and window center were input into training to provide more information. Furthermore, we built a multi-model fusion strategy to take multiple diagnostic results from different models into account,

which further improved the final detection performance. This method won the Global Data Intelligence Competition (2019 Tianchi Competition- Digital Human Race).

Artificial Intelligence (AI) method in imaging analysis: Accuracy of AI system

We applied 3 methods to guarantee the segmentation of lung lobes and lesions. Firstly, as indicated in Wang et al. [1], the proposed method achieved a dice of 0.97 in the HOUSE dataset (easy) and 0.89 in the LOLA dataset (hard) in terms of lung lobe segmentation, which demonstrated its effectiveness. There are a number of cases in the LOLA dataset where abnormalities presented by a large extent. Moreover, common lobe Dice loss and boundary Dice loss (in the auxiliary task of detecting lobar boundaries) were used. Secondly, in the aspect of lesions recognition, the model was trained with annotations labeled by senior radiologists. The boundary Dice loss was also used to enhance the boundary recognition. Thirdly, the segmentation result of cases in this study was checked by a radiologist (Y.C.W) with 10 years of experience in chest imaging. (the detailed flowchart of the check progress and possibly some quantitive evaluation)

Evaluated on the internal testing datasets, it achieved sensitivities of 90.1% and 90.3% for consolidation and ground-glass opacity detection with 1.8 and 1.4 false positives per scan separately. The lesion segmentation accuracy (measured by DICE coefficient) was 0.82. The pulmonary lobe segmentation achieved a dice of 0.97 in the HOUSE dataset (easy) and 0.89 in the LOLA dataset (hard).

[1] Wang XQ., Zhang QY., Zhou Z., Wang YZ., Yu YZ., Evaluating Multi-class Segmentation Errors with Anatomical Prior, IEEE International Symposium on Biomedical Imaging, 2020.

Table S1 Computed tomography (CT) parameters applied in study

Center	CT Manufacturer	Slice thickness (mm)	Matrix	KV	No. of scans (n = 446)[a]
1	UIH	1.5	512×512	120	29
2	SIEMENS	1	512×512	120	1
3	SIEMENS	1	512×512	120	3
4	Philips	1/3	512×512	120	76
5	SIEMENS	1.5	512×512	120	6
6	GE MEDICAL SYSTEMS	1.25	512×512	120	1
7	GE MEDICAL SYSTEMS	1.25	512×512	120	11
8	Philips	1	1024×1024	120	97
	TOSHIBA	1	512×512	120	
9	GE MEDICAL SYSTEMS	1.25	512×512	120	18
10	GE MEDICAL SYSTEMS	5	512×512	120	73
11	GE MEDICAL SYSTEMS	1.25	512×512	120	8
12	SIEMENS	1	512×512	120	10
13	SIEMENS	1	512×512	120	50
14	GE MEDICAL SYSTEMS	1.25	512×512	120	2
15	GE MEDICAL SYSTEMS	1.25	512×512	120	19
16	GE MEDICAL SYSTEMS	1.25	512×512	120	9
17	GE MEDICAL SYSTEMS	1.25/0.625	512×512	120	8
18	TOSHIBA	1	512×512	120	5
19	UIH	2	512×512	120	1
20	TOSHIBA	1/5	512×512	120	10
21	NMD	1.5/7	512×512	120	9

a Including 25 patients < 18 years.
